# Tumor Necrosis Factor Receptor-Associated Factor 4 Is a Dynamic Tight Junction-Related Shuttle Protein Involved in Epithelium Homeostasis

**DOI:** 10.1371/journal.pone.0003518

**Published:** 2008-10-27

**Authors:** Valérie Kédinger, Fabien Alpy, Aurélie Baguet, Myriam Polette, Isabelle Stoll, Marie-Pierre Chenard, Catherine Tomasetto, Marie-Christine Rio

**Affiliations:** 1 Institut de Génétique et de Biologie Moléculaire et Cellulaire (IGBMC), Department of Cancer Biology, CNRS UMR 7104 / INSERM U 596 / Université Louis Pasteur, Illkirch, France; 2 INSERM UMR-S 514, Laboratoire Pol Bouin, Hôpital Maison Blanche - C.H.U., Reims, France; 3 Service d'Anatomie Pathologique Générale, Centre Hospitalier Universitaire de Hautepierre, Strasbourg, France; University of Birmingham, United Kingdom

## Abstract

**Background:**

Despite numerous in vivo evidences that Tumor Necrosis Factor Receptor-Associated Factor 4 (TRAF4) plays a key biological function, how it works at the cellular and molecular level remains elusive.

**Methodology/Principal Findings:**

In the present study, we show using immunofluorescence and immuohistochemistry that TRAF4 is a novel player at the tight junctions (TJs). TRAF4 is connected to assembled TJs in confluent epithelial cells, but accumulates in the cytoplasm and/or nucleus when TJs are open in isolated cells or EGTA-treated confluent cells. In vivo, TRAF4 is consistently found at TJs in normal human mammary epithelia as well as in well-differentiated in situ carcinomas. In contrast, TRAF4 is never localized at the plasma membrane of poorly-differentiated invasive carcinomas devoid of correct TJs, but is observed in the cytoplasm and/or nucleus of the cancer cells. Moreover, TRAF4 TJ subcellular localization is remarkably dynamic. Fluorescence recovery after photobleaching (FRAP) experiments show that TRAF4 is highly mobile and shuttles between TJs and the cytoplasm. Finally, we show that intracellular TRAF4 potentiates ERK1/2 phosphorylation in proliferating HeLa cells, an epithelial cell line known to be devoid of TJs.

**Conclusions/Significance:**

Collectively, our data strongly support the new concept of TJs as a dynamic structure. Moreover, our results implicate TRAF4 in one of the emerging TJ-dependent signaling pathways that responds to cell polarity by regulating the cell proliferation/differentiation balance, and subsequently epithelium homeostasis. Drastic phenotypes or lethality in TRAF4-deficient mice and drosophila strongly argue in favor of such a function.

## Introduction

The compartmentalization-oriented architecture of the epithelia is a basic property of higher life forms. As cells assemble to form tissues, the organization of the plasma membrane becomes increasingly complex. Epithelial cells interact with each other via specialized cellular junctions that are critical for the normal development and function of the epithelia (reviewed in [1], [2]). They include adherens junctions (AJ), desmosomes and tight junctions (TJ). TJs encircle cells at the apical end of the lateral membrane to form a paracellular diffusion barrier that regulates epithelial permeability, and an intramembrane diffusion barrier which restricts the apico-basolateral diffusion of membrane components. There are a plethora of mechanisms that control or are controlled by TJs. Thus, TJs have been shown to contribute to epithelial biogenesis and function by regulating cell proliferation, differentiation and polarisation. Moreover, it has been proposed that the assembly state of TJs acts as a sensor for cell density. This implies that TJs can regulate downstream signaling pathways via a multiprotein complex network that constitute the TJ plaque, a process just beginning to be understood [3].

The Tumor Necrosis Factor Receptor-Associated Factor family is composed of 6 members that share a TRAF domain (TRAF1 to TRAF6). They are adaptor/scaffold molecules that mainly interact with members of the interleukin-receptor (ILRs) or tumor necrosis factor-receptor (TNFRs) families and exhibit functions mostly in the immune system [4], [5]. However, TRAF4 only weakly interacts with 3 members of TNF-R family, namely the p75 neurotrophin receptor (p75-NGFR), the lymphotoxin-beta receptor (LTâR), and the glucocorticoid-induced TNF-R (GITR), and its involvement in immune system remains elusive (reviewed in [6]). These data suggest that TRAF4 might rather be implicated in other biological processes. Different lines of evidence indicate that TRAF4 plays a key function. Indeed, mouse TRAF4 deficiency is embryonic lethal in approximately one third of the homozygote mutants. Surviving animals manifest numerous alterations, including important malformations in the trachea and the axial skeleton (ribs, sternum, tail), and defects in the neural tube closure giving rise to spina bifida phenotypes [7]. Similarly, a null allele for DTRAF1, the drosophila homologue of TRAF4, is embryonic lethal for drosophila. DTRAF1 is indispensable for the development of imaginal eye discs and the formation of a correct photosensory neuronal array in the brain hemisphere [8]. Moreover, in the adult, constitutive basal level of TRAF4 expression is observed in most tissues, suggesting a generic biological function for this protein. Finally, TRAF4 has also been observed to be overexpressed in various human cancers [9], [10].

Here, we show in vitro and in vivo that TRAF4 functions at the discrete TJ submembrane domain. Indeed, TRAF4 is observed at the TJ multiprotein complex areas. Moreover, fluorescence recovery after photobleaching (FRAP) analysis show that association of TRAF4 with the TJ is not a stable event but a highly dynamic process. Thus, TRAF4 is a shuttling protein. These results prompted us to investigate the ability of TRAF4 to activate the ERK1/2 MAP kinase and Akt signaling pathways which have been shown to be down-regulated by functional TJs. When not associated with TJs, TRAF4 acts to increase ERK1/2 phosphorylation in proliferating but not confluent cells. Collectively, our results allow us to assign a biological function to the TRAF4 scaffold protein in favoring the ERK1/2 signalling pathway in response to cell polarity damage.

## Results

### Endogenous TRAF4 is found in the plasma membrane, cytoplasmic and nuclear fractions of normal/immortalized human MCF10A confluent cells

**Figure 1:**
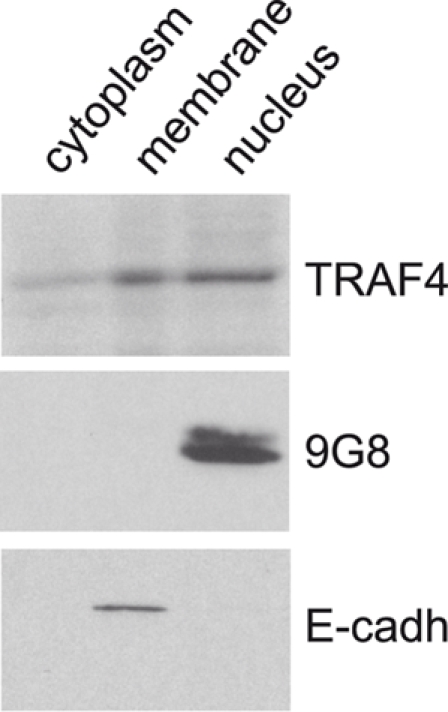
Subcellular localization of endogenous TRAF4 in fractionated extracts of MCF10A cells. Protein fractionation from confluent normal/immortalized MCF10A cells was performed using the ProteoExtract Subcellular Proteome Extraction kit in order to separate cytoplasmic, membrane, and nuclear proteins. These fractions were analyzed by western blot using anti-E-cadherin, anti-9G8 and 2H1 anti-TRAF4 antibodies. TRAF4 is observed in all fractions whereas the membrane specific E-cadherin and nucleus specific 9G8 are only seen in membrane and nuclear fraction, as expected.

To date, the sub-cellular TRAF4 localization pattern remains debated. In in vitro experiments, TRAF4 has been reported to localize at the cell membrane, in the cytoplasm, in perinuclear areas or in the nuclei (reviewed in [6]). In carcinomas, its localization is cytoplasmic and/or nuclear [10]. More importantly, very few data concerning endogenous TRAF4 sub-cellular localization under normal conditions are available. In order to address this question, we performed a stringent fractionation assay (see material and methods) to separate the membrane, cytoplasmic and nuclear compartments from confluent MCF10A cells, derived from human normal/immortalized mammary epithelial cells. Extracts were loaded on SDS-PAGE and the presence of endogenous TRAF4 was tested by Western blot analysis using the 2H1 mouse monoclonal antibody as previously described [7]. Antibodies specific for E-cadherin, a marker for plasma membrane and cytoskeleton [11], and the serine/arginine-rich (SR) protein 9G8, a splicing factor that is mainly observed in nuclei [12] were used as controls. TRAF4 was observed in all fractions (Figure 1). As expected, E-cadherin and 9G8 were detected only in the membrane and nuclear extracts, respectively.

Thus, endogenous TRAF4 is associated with the plasma membrane, cytoplasmic and nuclear sub-cellular fractions under normal conditions.

### The TRAF domain is responsible for TRAF4 plasma membrane and nuclear addressing

**Figure 2:**
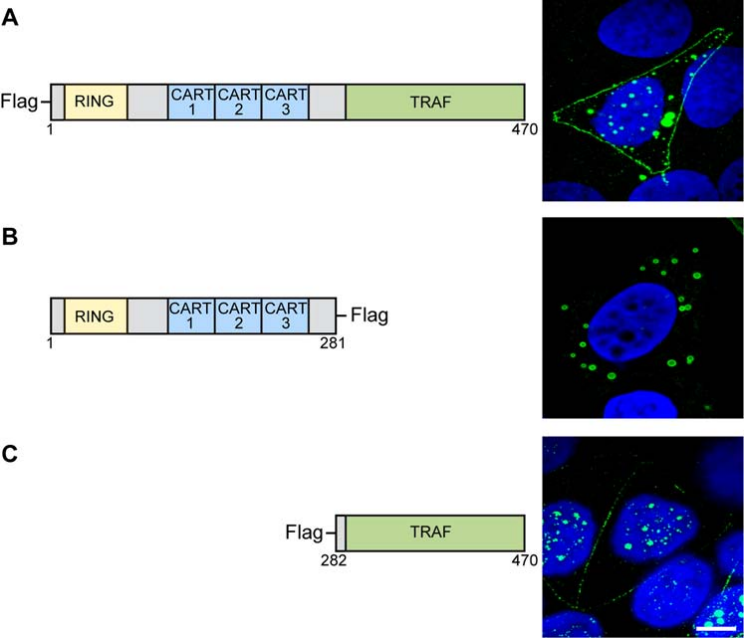
Plasma membrane, cytoplasm and nucleus addressing of TRAF4 deletion mutants. Left part: Schematic representation of the 3 constructs coupled to Flag. A: full-length TRAF4 (AA1-470), B (AA1-281) and C (AA282-470). Right part of the figure represents image projection of several confocal sections. Mutant C containing the TRAF domain only was addressed to the plasma membrane and nucleus, like the full-length TRAF4 protein, but was absent from the cytoplasm. In contrast, mutant B devoid of the TRAF domain was only addressed to the cytoplasm. AA: amino acid.

We then investigated which conserved domains within TRAF4 could be responsible for these localizations. The TRAF4 protein contains 1 RING, 3 CARTs and 1 TRAF domains [9]. Full-length TRAF4 (AA1-AA470, construct A) and 2 deletion mutants -mutant B (AA1–AA281) corresponding to the amino-terminal part of TRAF4 containing the RING and the 3 CART domains, and mutant C (AA282–AA470) corresponding to the carboxy-terminal part of TRAF4 containing the TRAF domain - were fused to the Flag epitope (Figure 2, left part). These constructs were transiently transfected in various cell lines and the sub-cellular localization of their products were evaluated using the anti-flag antibody using confocal microscopy (Figure 2, right part). Full-length TRAF4 was located at the plasma membrane, cytoplasm and nucleus, indicating that the 3 TRAF4 localizations are not exclusive, at least in transfected cells. Mutant B devoid of the TRAF domain was only found in the cytoplasm. In contrast, mutant C which contains only the TRAF domain showed membrane and nuclear localization, but was absent from the cytoplasm.

Collectively, these results indicate that the TRAF domain is required to address TRAF4 to the plasma membrane and the nucleus.

### Cell-cell contacts govern the membrane localization of TRAF4

**Figure 3:**
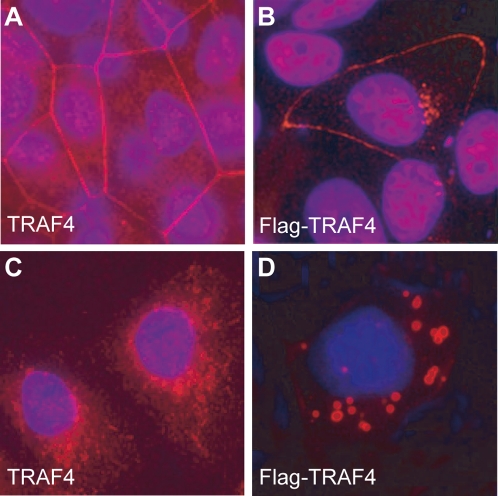
TRAF4 localization depending on cell density. IF analyses of the localization of endogenous TRAF4 (A and C) or transiently transfected Flag-TRAF4 (B and D) in confluent (A and B) or sparsely populated (C and D) MCF10A cells. Cells were fixed, permeabilized and stained using 2H1 anti-TRAF4 antibody (A and C) or an anti-Flag antibody (B and D), respectively. Nuclei were stained using Hoechst-33258 (blue). Localization of endogenous and exogenous TRAF4 at the plasma membrane is lost in sparse cells, and TRAF4 is mostly cytoplasmic. A weak nuclear staining is observed in cells expressing Flag-TRAF4. Thus, TRAF4 is only present at the plasma membrane when cells are confluent. Analyses were performed using a fluorescence microscope (Leica DMLB 30T, Leica Micro system, Wetzlar Germany). Magnification 63×.

To visualize endogenous TRAF4 in cells, we first performed immunofluorescence analysis (IF) of confluent MCF10A cells using the 2H1 antibody, using classical fluorescence microscopy. TRAF4 was observed mainly in the cytoplasm and at the plasma membrane (Figure 3 A). In parallel, we performed similar experiments using MCF10A cells transiently transfected with a full-lenght TRAF4 fused to the Flag epitope at its amino-terminal (Flag-TRAF4 ; construct A). The pattern of Flag-TRAF4 was similar to that of endogeneous TRAF4 with the addition of a low nuclear staining (Figure 3 B). We then studied TRAF4 localization in isolated MCF10A cells present in non-confluent culture. In this case, neither endogenous nor transfected Flag-TRAF4 proteins were found at the plasma membrane (Figure 3 C and D), showing that cell-cell contacts are required for the recruitment of TRAF4 to the membrane. Moreover, a stronger cytoplasmic patched staining was observed in Flag-TRAF4 transfected cells (Figure 3D). This is probably due to excess TRAF4 protein, since similar staining was observed in vivo in human invasive carcinomas overexpressing TRAF4 (see below and [9], [10]).

Thus, TRAF4 localizes to the plasma membrane only when cells are confluent suggesting that TRAF4 function might be related to intercellular junctions.

### TRAF4 is related to the TJs

**Figure 4:**
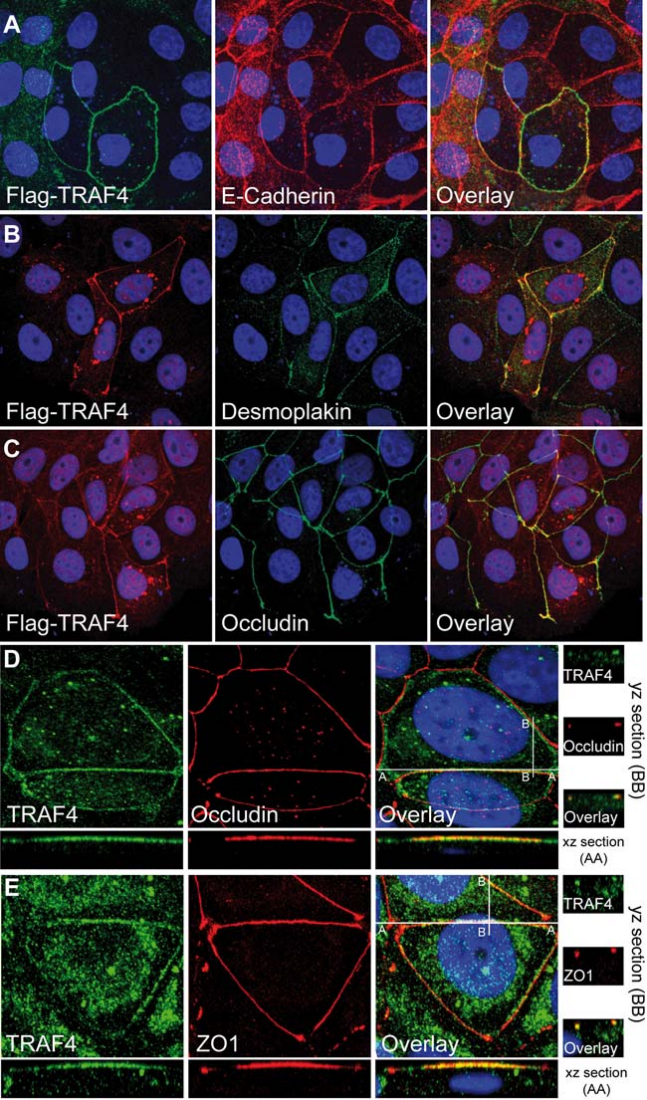
TRAF4 localization at cell-cell junctions. (A–C) : MCF7 cells transiently transfected with Flag-TRAF4 were subjected to IF experiments. Co-staining was performed using an anti-Flag antibody in combination with either anti-E-cadherin (panel A), anti-desmoplakin (panel B), or anti-occludin (panel C) antibodies specific for AJs, desmosomes, and TJs, respectively. A partial colocalization of TRAF4 with the 3 proteins was observed, the highest overlapping rate was observed with occludin. (D) : Co-staining of endogenous TRAF4 and occludin in MCF7 cells using the 2H1 anti-TRAF4 and anti-occludin antibodies. xz- and yz-scans show the expected linear and punctual localization of human TJ-related protein occludin (red) in sections of 0.4 µm from the apical pole of the cell. TRAF4 (green) exhibited similar staining. (E) : Co-staining of endogenous TRAF4 (green) and TJ-related protein ZO1 (red) in MCF7 cells using the 2H1 anti-TRAF4 and anti-ZO1 antibodies. There is a perfect colocalization of these proteins. Collectively, these data show that, at the plasma membrane, TRAF4 is restricted to the TJs. For all experiments, nuclei were stained using Hoechst-33258 (blue), and microscopy analysis was performed using a Zeiss Axiovert 100 M confocal laser scanning microscope equipped with LSM5 Pascal (Jena, Germany). The figure represents image projection of several confocal sections. Magnification : A–C , 40×; D and E, 63×.

To determine if TRAF4 binds a particular junction, we performed an extensive colocalization study of TRAF4 with markers of cell-cell junctions. Confluent monolayers of MCF7 cells (well-differentiated human breast cancer cells) were tested for the presence of transiently-transfected human Flag-TRAF4 at AJs (Figure 4 A), desmosomes (Figure 4 B) and TJs (Figure 4 C), as identified by their respective markers E-cadherin, desmoplakin and occludin using appropriate antibodies and fluorescence confocal microscopy. E-cadherin, desmoplakin and occludin staining partially overlapped. This is consistent with reports showing that AJs can be concentrated close to TJs, or be distributed over the entire lateral membrane like desmosomes [13], [14]. We observed that Flag-TRAF4 was not randomly distributed along the plasma membrane, but partially colocalized with the E-cadherin, desmoplakin and occludin. The best colocalization pattern was observed with occludin (Figure 4 C and data not shown), suggesting that TRAF4 localizes to TJs. Since these experiments evaluated only exogenous Flag-TRAF4, we checked for sub-membrane localization of endogenous TRAF4. Using the 2H1 antibody on human MCF7 cells, IF staining showed that endogenous TRAF4 colocalized with endogenous occludin at the TJs (Figure 4 D). Both yz- and xz-scans showed respectively similar punctual and linear staining for TRAF4 and occludin at the apical part of the lateral plasma membrane of confluent cells, typical of TJs,

TJs are composed of a multiprotein complex which includes transmembrane proteins (ie : occludin), and supra-membrane proteins (ie : zonula occludens 1 (ZO1) and ZO2), recruited by the former and which constitute the cytoplasmic plaque [15]. Localization of TRAF4 at the TJ was further confirmed in colocalization experiments with ZO1 in MCF7 cells. IF staining showed that endogenous TRAF4 colocalized with endogenous ZO1 at the TJs. As expected, both xz- and yz-scans showed similar staining for TRAF4 and ZO1 at the TJs (Figure 4 E). Finally, similar results were obtained for 2 normal epithelial cell lines: in transient transfection experiments using Madin-Darby Canine Kidney (MDCK) cells which are known to become polarized at confluency [16], and in human MCF10A cells (data not shown).

Thus, in several epithelial cell types, normal or malignant, we observed that TRAF4 is not homogeneously distributed along the plasma membrane but accumulates at the TJs of contacting neighboring cells.

### TRAF4 is present at the TJ multiprotein complex in normal human epithelia

**Figure 5:**
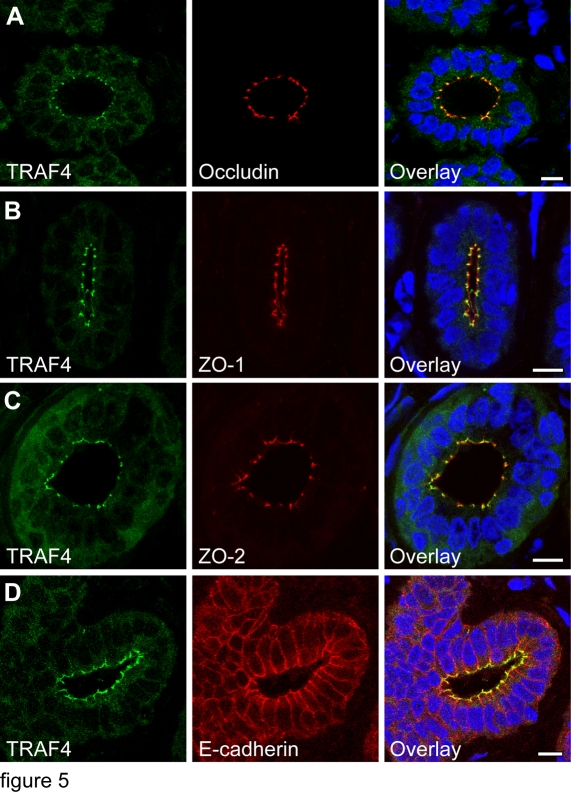
TRAF4 localization at the plasma membrane of normal human mammary epithelium. (A–D): TRAF4 (green) and occludin (A, red), ZO1 (B, red), ZO2 (C, red) or E-cadherin (D, red) were co-labelled on paraffin embedded sections of human normal breast ducts. Images are confocal transverse sections. Nuclei were stained using Hoechst-33258 (blue). Strong focal staining at the apical part of the plasma membrane which correspond to TJs is observed for TRAF4, occludin, ZO1 and ZO2. In contrast, E-cadherin localizes along the entire lateral plasma membrane. Thus, TRAF4 is seen at the TJs sealing neighboring cells in normal mammary ducts. TRAF4 cytoplasmic staining is also observed. Scale bars: 10 µm.

We studied the in vivo submembrane distribution of endogenous TRAF4 in the polarized epithelial cells that form the normal human mammary epithelium. The colocalization of ZO1, ZO2 and occludin, and E-cadherin were performed on adjacent serial mammary gland sections using immunofluorescence (IF) confocal microscopy to visualize TJs and AJs respectively (Figure 5 A–D). The TRAF4 pattern was similar to those of ZO1, ZO2 and occludin. Thus, on mammary duct transversal sections, they showed punctate staining at epithelial cell-cell contacts, in the apical part of the lateral membrane. The other parts of the plasma membrane were devoid of staining (Figure 5 A–C). By contrast, E-cadherin extended to the entire lateral plasma membrane (Figure 5 D). The colocalization of TRAF4 with ZO1, ZO2, and most notably occludin, a transmembrane protein specific of TJs, indicates that TRAF4 is present at the TJ multiprotein plaque.

Thus, in vivo, TRAF4 is not randomly distributed along the plasma membrane but is restricted to the TJ submembrane domain in normal human epithelia.

### TRAF4 is addressed to assembled TJs

**Figure 6:**
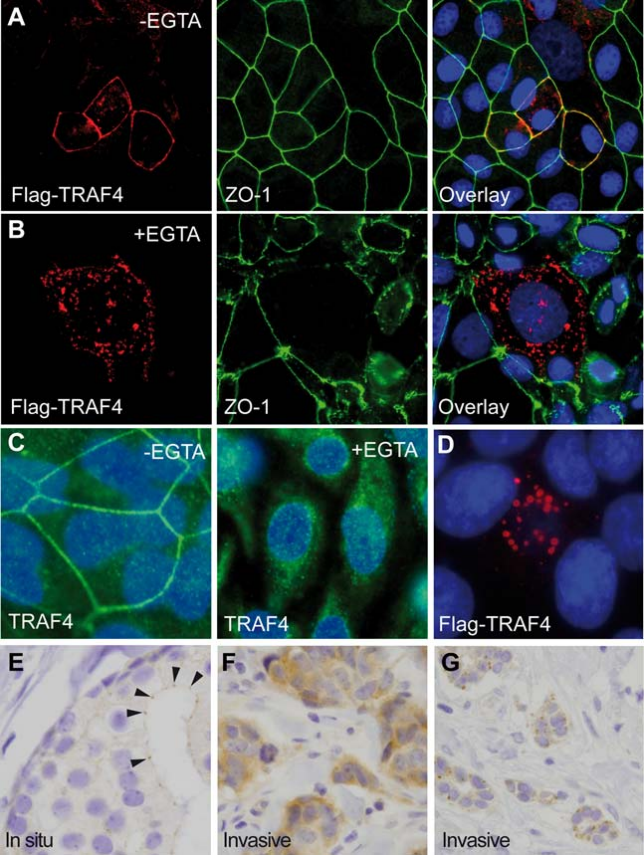
TRAF4 localization in disrupted TJs. (A–B): Chemical disruption of TJs : Confluent MCF7 cells transiently transfected with Flag-TRAF4 were treated for 2 hours with (B) or without (A) EGTA. Cells were fixed and stained using anti-Flag and anti-ZO1 antibodies. After EGTA incubation, most TJs were disrupted with concomitant loss of localized membrane staining for TRAF4. Moreover, TRAF4 and ZO1 no longer colocalize in the absence of TJs since, although it loses its honeycomb pattern, ZO1 remains partially associated with the plasma membrane whereas TRAF4 becomes cytoplasmic. (C) : similar experiments were performed using confluent MCF10A and 2H1 anti-TRAF4 antibody. While untreated cells show a characteristic honeycomb staining pattern for TRAF4 (left part), endogenous TRAF4 membrane localization is lost after EGTA-treatment (right part). For experiments (A–C), nuclei were stained using Hoechst-33258 (blue), and microscopy analysis was performed using a fluorescence microscope (Leica DMLB 30T, Leica Micro system, Wetzlar Germany) (C, magnification 63×), or a Zeiss Axiovert 100 M confocal laser scanning microscope equipped with LSM5 Pascal (Jena, Germany) (A and B, magnification 40×). (D): Confluent HeLa cells transiently transfected with Flag-TRAF4 (red). Note the absence of TRAF4 at the plasma membrane (magnification 80×). (E) : IH analysis of endogenous TRAF4 expression in human well-differentiated in situ breast carcinomas. 2H1 anti-TRAF4 antibody was used on paraffin embedded sections. Focal staining is observed in the epithelial structures at the apical part of the lateral sides of the plasma membrane of the epithelial cells which correspond to TJs (arrow heads). Thus, TRAF4 remains at the TJs sealing neighboring cells in well-differentiated in situ carcinomas. (F,G): Similar IH analysis of endogenous TRAF4 expression in human invasive breast carcinomas known to be devoid of functional TJs [10]. No TRAF4 staining was observed at the plasma membrane but strong diffuse or patched staining was seen in the cytoplasm. Thus, assembled TJs are required for TRAF4 plasma membrane localization. Magnification: E, ×200; F and G, ×150.

Cell-cell junctions are primordial players in tissue differentiation and homeostasis. Epithelial tumor progression involves a series of cumulative alterations. Invasive and migratory properties should notably be acquired, enabling cells to pass from a sessile to a motile phenotype and to ultimately metastasize. During this process, disruption of cell-cell adhesion and loss of functional junctions is a major event. Previous studies of invasive tumors did not report TRAF4 plasma membrane localization, but cytoplasmic in a large majority of cases and/or nuclear localization in around 20% of cases [10]. Thus, we hypothesized that TRAF4 localizes to the plasma membrane only when TJs are correctly assembled. To test this hypothesis we forced TJ alteration in confluent MCF7 cells transiently transfected with human Flag-TRAF4 grown in the presence of EGTA, a Ca2+ chelating agent that disrupts TJs [17]. In these cells, TRAF4 became cytoplasmic and nuclear (Figure 6 A and B). By contrast, in the same condition, ZO1 remained associated with the plasma membrane although its honeycomb pattern was lost (Figure 6 B), similar to previous reports [17]. Similarly, in EGTA-treated confluent monolayer of MCF10A cells, endogenous TRAF4 staining became mainly cytoplasmic and nuclear, and no plasma membrane staining was observed (Figure 6 C, right part). As an additional control, we analyzed the localization of TRAF4 in HeLa cells which are devoid of TJs, even when confluent [18]. In this cell line, TRAF4 was never observed at the plasma membrane, even when overexpressing Flag-TRAF4 (Figure 6 D). Finally, under optical microscopy, in in situ breast primary tumors exhibiting well-differentiated features, although the epithelial cells are malignant we observed a TRAF4 TJ pattern at the remaining cell-cell contacts (Figure 6 E), similar to that shown in normal epithelium (Figure 5), whereas invasive carcinomas are totally devoid of such staining (Figures 6 F and G), but exhibit cytoplasmic and/or nuclear staining [9], [10].

Collectively, our results indicate that TRAF4 is connected to the plasma membrane with assembled TJs, suggesting a TJ-related function for this protein.

### TRAF4 connection at the TJs is higly dynamic

**Figure 7:**
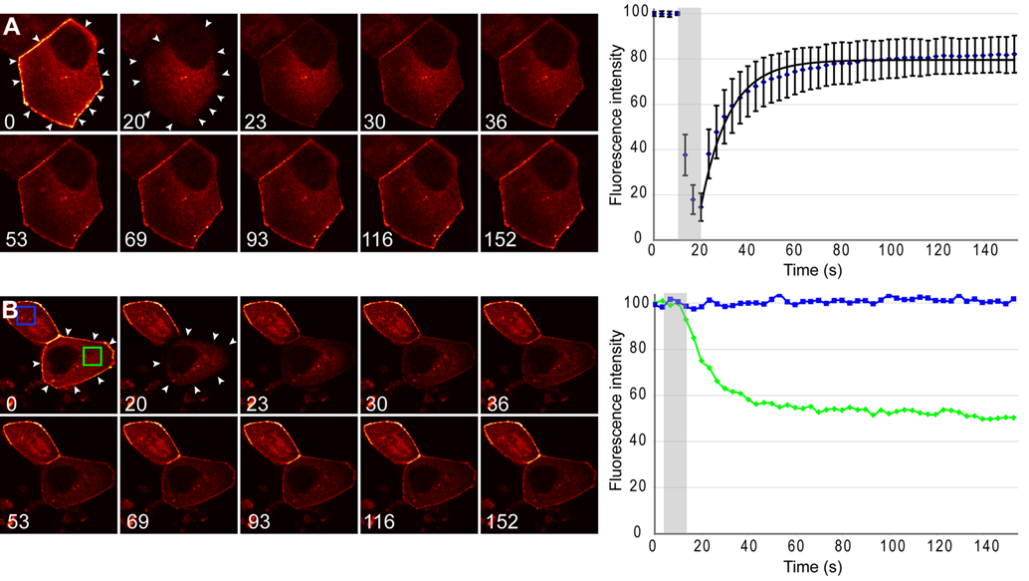
FRAP analysis of TRAF4 mobility into TJs. MDCK cells transfected with EYFP-TRAF4 for 72 hours were analyzed by FRAP. (A): One representative cell expressing EYFP-TRAF4 is shown at different time points (time in seconds). All TJs were bleached and pictures were taken every 3.3 s. The bleach zone is highlighted with white arrows. Graphical representation of fluorescence recovery pattern is shown on the right (n = 14). The bleach period is highlighted by the gray rectangle. A fitted curve is shown. Calculated half recovery time (t1/2) is 8.3 s and the mobile fraction is 76%. (B): cytoplasmic fluorescence decay in 1 bleached (lower) and 1 unbleached (upper) EYFP-TRAF4 expressing cell. Fluorescence intensity was measured in similar cytoplasmic areas (blue and green squares) of the unbleached and bleached cell respectively. The fluorescence intensity over time is shown for these two areas on the right. While the fluorescence intensity remains constant in the unbleached cell, it decreases over time in the bleached cell, showing that cytoplasmic EYFP-TRAF4 molecules are mobilized to the bleached area during recovery. Thus TRAF4 shuttles very rapidly between the cytoplasm and TJs.

FRAP studies offer new insight into basic biological mechanisms [19], as they permit the measurement of force-dependent alterations in molecular binding and unbinding of individual proteins in situ in the physical context of the living cytoplasm [20]. The combination of time-lapse imaging with FRAP enables the analysis of the kinetic properties of a given protein in living cells. As photobleaching is an irreversible process, fluorescence recovery comes from proteins already present in the cell. Thus, the recovery kinetics are dependent on the mobility of the protein of interest. Slow recovery indicates low mobility. Thus, we ask whether TJ association of TRAF4 is stable, or whether TRAF4 can dynamically move in and out of TJs. We therefore generated a EYFP tagged TRAF4 fluorescent protein (EYFP-TRAF4) to test the kinetics of TRAF4 recruitment to the TJs using the FRAP technique. MDCK cells were transfected with EYFP-TRAF4, allowed to polarize during 72 hours after transfection and then subjected to FRAP. In order to avoid visualization of intramembrane protein movements, all of the TJs of the cell were bleached. FRAP experiments showed that EYFP-TRAF4 rapidly recovers within the bleached area. Thus 50% recovery (t1/2) is observed in 8.3 seconds indicating that TRAF4 is a highly mobile protein. The recovery curve reached a plateau at 75% of the initial fluorescence of the pre-bleached area, indicating that only a small proportion of the TRAF4 protein was immobile (immobile fraction = 24%) (Figure 7 A). The cytoplasmic fluorescence intensity variations were also monitored in either bleached or unbleached cells. Interestingly, we showed that in parallel to the gain of fluorescence in TJs after the bleach, the fluorescence of the cytoplasm decreased. These results indicate an exchange of TRAF4 molecules between the cytoplasm and the TJs (Figure 7 B, and Movies S1 and S2). The cytoplasmic fluorescence intensity remains constant in the unbleached cells.

These experiments reveal that the TJ association of TRAF4 in epithelial cells is highly dynamic and that TRAF4 shuttles between TJs and cytoplasm.

### Intracellular TRAF4 potentiates ERK1/2 MAPKinase mediated signals in proliferating cells

**Figure 8:**
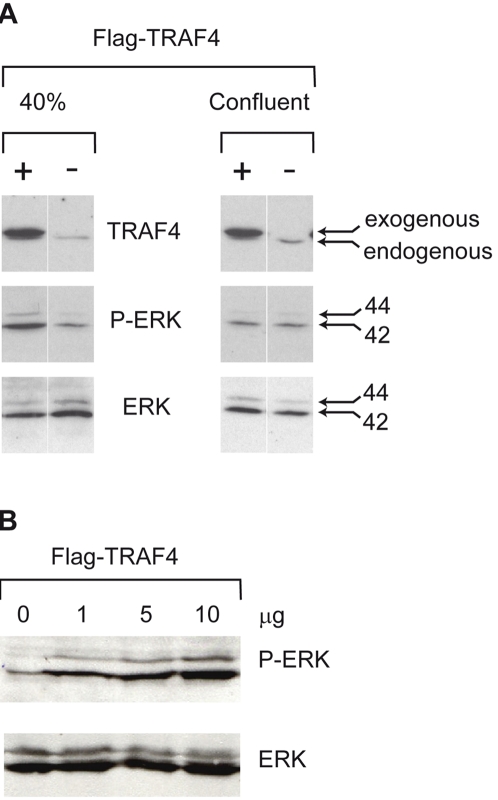
Effect of cytoplasmic TRAF4 on ERK signaling pathway. (A): 40% and 100% confluent HeLa cells that never form TJs (Figure 6 D) were transfected with the Flag-TRAF4 expression vector (2.5 µg) or an empty vector (2.5 µg). Twenty-four hours after transfection, proteins were extracted, and analyzed by immunoblotting with antibody for phospho-ERK1/2. Total amounts of ERK1/2 were evaluated by reprobing the blots with pan-ERK antibody. TRAF4 expression was tested using 2H1 anti-TRAF4 antibody. Activation of ERK1/2 is dependent on cell confluency. (B): a similar experiment in non-confluent cell condition using increasing amounts of the Flag-TRAF4 expression vector (1 µg, 5 µg or 10 µg). Activation of ERK1/2 is dependent on the amounts of transfected Flag-TRAF4. (A and B): a representative western blot from four independent experiments is shown. Thus, TRAF4 favors ERK1/2 MAPK activation in proliferating HeLa cells.

Previous studies have associated TJ function with the extracellular signal-regulated kinase 1 and 2 (ERK1/2) mitogen-activated protein kinase (MAPK) and Akt/PKB (protein kinase B) pathways [3], [21]. It has been proposed that TJs may decrease the activity of these pathways most probably via recruited proteins. We hypothesized that TRAF4 might participate in this process when not recruited to the TJs. However, this was particularly difficult to test due to the highly dynamic manner of TRAF4 shuttling. Since these pathways participate in the regulation of cell proliferation/differentiation [22], we tested the ability of overexpressed Flag-TRAF4 (2.5 µg) to increase ERK1/2 and Akt signalling at 2 different cell densities: 40% (proliferating cells) and 100% confluent (resting cells). Phosphorylation of ERK1/2 and Akt was tested by Western blots using antibodies specifically directed against the phosphorylated forms of ERK1/2 and Akt. Total ERK1/2 and Akt were detected using specific pan-antibodies. The Flag-TRAF4 was visualized using the 2H1 anti-TRAF4 antibody. As it is very difficult to obtain epithelial cells completely devoid of TJs, even in low density culture conditions (these cells frequently form ilots), we used HeLa cells that, although epithelial in origin, are devoid of TJs [18] and never show TRAF4 at their plasma membrane, even when overexpressed (Figure 6 D). This allowed us to discard any possibility of TRAF4 association with TJs, and to test the effect of intracellular TRAF4. Akt was not phosphorylated in all cases (data not shown). However, we observed increased phosphorylation of ERK1/2 in the presence of TRAF4 in low-density cells, but not in confluent cells (Figure 8 A). When we transfected increasing Flag-TRAF4 amounts (1, 5 and 10 µg) into low-density cells, ERK1/2 phosphorylation was enhanced in a TRAF4 dose-dependent manner (Figure 8 B). These results indicate that, although unable to induce ERK1/2 phosphorylation in resting cells, TRAF4 potentiates the ERK1/2 signaling pathway in proliferating HeLa cells.

Thus, when not associated with TJs, TRAF4 increases the ERK1/2 MAPKinase signaling pathway in non-confluent and proliferating cells.

## Discussion

While other TRAFs have been mostly implicated in the immune system as adaptor proteins of TNF-R and IL-R [4], [5], the TRAF4 biological function has remained elusive [6]. Our present data provide in vitro and in vivo evidence that TRAF4 function is related to epithelial cell polarity and epithelium homeostasis.

Using several cell lines and human breast tissues, we showed that TRAF4 localizes to a remarkable subdomain of the plasma membrane corresponding to multiprotein complexes that form TJs. TJs are intercellular adhesion structures that are indispensable for correct epithelium organization. TJs assemble when epithelial cells reach high cell densities and adhere to each other, giving rise to well organized and differentiated functional polarized tissues. We showed that the TRAF domain of TRAF4 is indispensable for this localization. This particular sub-membrane localization of TRAF4 was unexpected as none of the putative TRAF4 partners identified until now corresponds to proteins already reported to be related to TJs [2], [23], [24]. Thus, using in vitro experiments, more than 20 putative TRAF4 partners have been identified. These include the p70S6K ser/thr kinase, Misshapen (Msn) and Pelle (the drosophila homologues of mammalian Nck-interacting kinase (NIK) and Interleukin-1 receptor-associated kinase (IRAK), respectively), the MAPKinase MEKK4, the p47phox adapter subunit of the NAD(P)H oxidase, nuclear proteases, apoptosis inhibitors…‥ [6]. The in vivo relevance of these TRAF4 partners has yet to be established.

Functional TJs are associated with well-differentiated non-proliferating cells. TJs are normally disrupted to allow cell division, tissue renewal and wound healing. We established that TRAF4 is only connected to assembled TJs. While TRAF4 is clearly associated with TJs in confluent cells, no TRAF4 staining remains at the cell plasma membrane in isolated cells nor in confluent cells after EGTA-induced TJ disruption. Similarly in vivo, TRAF4 is observed at the TJs sealing epithelial cells of normal mammary epithelium. Loss of cell-cell adhesion and TJ alteration occurs as epithelial tumors progress [25]. Moreover, fully formed TJs in high-density cells have been shown to function as suppressors of signaling pathways that stimulate proliferation and inhibit differentiation, thereby contributing to epithelium biogenesis and/or homeostasis [23]. Interestingly, aggressive malignant tumors never exhibit membrane TRAF4 staining [9], [10]. However, in in situ tumors which have conserved some structural epithelial organization and therefore TJs, this TRAF4 staining remains. Together these data indicate that TRAF4 is connected to assembled TJs, but not to scattered TJ proteins.

The visualization of proteins by IH reveals a steady-state picture with a defined distribution within the cell. However, protein mobility within a cell is of key importance since it is an essential prerequisite for numerous cellular functions [26]. We therefore analyzed TRAF4 TJ addressing in FRAP experiments to track intracellular events in living cells. Fluorescence recovery occurs very rapidly, indicating that TRAF4 is a highly mobile protein that exchanges between membrane and intracellular pools. Moreover, cytoplasmic fluorescence decreased over time in the bleached cells strongly supporting a shuttling function for TRAF4 between the TJs and the cytoplasm. These data are consistent with the recent report showing that the TJ protein complex undergoes rapid and continuous molecular remodeling [27]. Indeed, until now, the TJ was widely viewed as a static structure. Our data strongly support the new concept of TJ as a dynamic structure.

There is a growing evidence that TJ-related protein function can depend on their dual localization both at TJs and nucleus [26]. Most of them have been linked to the regulation of transcription or to RNA processing [23]. ZO1-associated nucleic acid-binding protein (ZONAB), a transcription factor required for G1/S phase progression, is TJ-associated in confluent cell cultures but accumulates in nucleus when cells are dispersed [13]. Thus, ZONAB sequestration by functional TJs reduces cell proliferation. ZO1 re-localization has been shown to be associated with induction of invasion in pancreatic cancer cells [28], or induction of matrix metalloproteinase 14 transcription [29]. ZO2 interacts with the nuclear ribonucleoprotein (hnRNP) Scaffold Attachment Factor B (SAF-B) in proliferating cells [30]. The mechanism of their shuttling is not clearly established. Since TRAF4 is also observed in the nucleus, most notably in invasive malignant tumors known to be devoid of TJs, a similar shuttling between TJs and nucleus cannot be excluded. A shuttling function for TRAF4 between TJs and nucleus is compatible with its absence at the plasma membrane and its nuclear accumulation in 20% of various types of cancer cells [10], and supports the idea that TRAF4 might contribute to maintaining tissue homeostasis by regulating proliferation/differentiation processes.

How TJ complexes actually signal and maintain cell polarization is still poorly understood. It has been proposed that the occludin TJ compound can directly interact with phosphatidylinositol-3 kinase (PI3K) to allow the phosphorylation of phosphatidylinositol 2 phosphate (PIP2) into PIP3, and link TJs to lipid signaling, and indirectly, to the Akt pathway [23]. However, TRAF4 overexpression does not induce Akt phosphorylation, indicating that TRAF4 is not involved in this pathway. TJs have also been reported to be connected to Raf-1 signaling since an overexpression of occludin reverses Raf-1-mediated cell transformation [21]. In numerous human cancers, there is a direct correlation between loss of TJs, deregulation of Ras signaling, and cancer progression and metastasis [23]. The best known effectors of Ras are the Raf kinases which stimulate cell cycle entry via ERK/MAPkinase activation, leading to anarchic cell proliferation in cancers. Interestingly, we showed in non-confluent epithelial HeLa cells devoid of TJs that TRAF4 overexpression increases ERK1/2 phosphorylation in a dose-dependent manner, indicating that cytoplasmic TRAF4 can enhance this MAPK signaling. Similar TRAF4 activation of ERK1/2 MAPK was recently reported in endothelial cells [31]. It is becoming more and more evident that ERK signaling cascade plays a role in the regulation of various cellular processes depending on cooperating mechanisms not fully understood to date (reviewed in [22]). In this context, TRAF4 might contribute to signaling events via the ERK pathway when it is not engaged at TJs. Such a function is consistent with the putative function of TRAF4 as an oncogene [10], [32], [33]. Thus, TRAF4 activity might be negatively regulated when connected to TJs. Since TRAF4 is never addressed to the plasma membrane in invasive carcinomas, it can be hypothesized that this function on ERK might be constitutively turn on in malignant conditions.

In conclusion, our collective results allow us to propose a cellular function for the TRAF4 scaffold protein in mediating plasma membrane dynamics in response to cell polarity damage to maintain cell proliferation/differentiation balance and therefore epithelium homeostasis. Such a function is consistent with the fact that TRAF4 TJ localization is lost in favor of the cytoplasm and/or nucleus in poorly-differentiated invasive tumors [10]. It is also consistent with the hypothesis of a generic function for TRAF4 in tissue proliferation/differentiation and organ ontogenesis during embryogenesis [6]. Indeed, it has been shown that TJ formation during development is critical for embryonic patterning and organization [34] while TRAF4-deficient mice [7] and drosophila present drastic phenotypes or lethality [8].

## Materials and Methods

### Cell culture

The normal/immortalized mammary epithelial MCF10A cell line was a kind gift of M. Polette [35]. The cancer epithelial cell lines MCF7 and HeLa, as well as the normal/immortalized canine epithelial cell line MDCK, are described and available at the American Type Culture Collection (ATCC, Rockville, MD). Cells were routinely maintained in our laboratory and cultured as recommended.

In order to disrupt TJs, Ca^2+^ chelation was performed by treating confluent MCF10A or MCF7 cells with 2.5 mM EGTA for 2 hours. EGTA was then removed and cells were fixed and stained using appropriate antibodies to visualize TRAF4, Flag-TRAF4 and ZO1.

### TRAF4 subcellular distribution

To examine the normal subcellular distribution of endogenous TRAF4, confluent MCF10A cells were fractionated using the ProteoExtract Subcellular Proteome Extraction kit (Calbiochem, La Jolla, CA). The proteins present in supernatant were quantified by the Bradford technique using a detergent-compatible protein assay (Bio-Rad), and equivalent total protein amounts were loaded on 10% SDS-PAGE gels, and electrotransferred to nitrocellulose sheets (Schleider and Schuell, Dassel, Germany). The membranes were blocked in PBS containing 3% fat-free milk and 0.1% Tween 20. The mouse monoclonal anti-TRAF4 2H1 antibody (1/5000; IGBMC, [7]), the rat monoclonal anti-E-cadherin antibody (1/400; Boehringer, Mannheim, Germany) and rabbit polyclonal anti-9G8 antibody (1/1000 ; kind gift of R. Gattoni, IGBMC, [12]) were used as primary antibodies. Horseradish peroxidase-conjugated AffiniPure goat anti-mouse, donkey anti-rat, or goat anti-rabbit (Jackson Immunoresearch, West Grove, PA) were used as secondary antibodies at dilution of 1/10000. Protein-antibody complexes were visualized by an enhanced chemiluminescence detection system (SuperSignal West Pico, Pierce, Pockford, IL).

### ERK and AKT analysis

To test the ability of TRAF4 to activate ERK1/2 and Akt, HeLa cells were plated in 100 mm dishes at low (40%) or high (100%) cell density and transiently transfected with appropriate amounts of the Flag-TRAF4 pRK5 expression vector, or empty vector as control, using the JetPEI™ transfection reagent (Polyplus Transfection, Illkirch, France) with 10 µg of total DNA. Forty-eight hours after transfection, cells were washed in PBS and cell lysis was performed by incubating the cells 15 minutes at 4°C in 150 µl of lysis buffer (50 mM Tris·HCl, pH 7.5, 150 mM NaCl, 1 mM EDTA, 1% Triton X-100; 1× protease inhibitor mixture and 1× phosphatase inhibitor cocktail). Cellular debris were removed by centrifugation at 10000×g for 10 minutes. Equivalent amounts of protein were loaded on 10% SDS-PAGE gel and analyzed by western blot using polyclonal rabbit anti-Phospho-ERK1/2 (1/1000), anti-ERK1/2 (1/1000), anti-Phospho-Akt (1/1000) or anti-Akt (1/1000) antibodies (Biotechnology, Santa-Cruz, CA).

### Cell immunofluorescence analysis

Five to ten thousand MCF7, MCF10A, HeLa or MDCK cells were plated on coverslips in 24-well plates and transfected using JetPEI™ transfection reagent (Polyplus Transfection, Illkirch, France) with 3 µg of the plasmids of interest. After 24 hours, cells were washed in PBS, fixed for 5 minutes at room temperature in 4% paraformaldehyde in PBS, and permeabilized 2 times for 10 minutes with 0.1% Triton X-100 in PBS. To visualize endogenous TRAF4 with 2H1 antibody, cells were fixed in 50% Methanol/50% Acetone for 15 minutes at −20°C; no permeabilization was needed in this case. After blocking with 1% BSA fraction V in PBS for 30 minutes, cells were incubated at room temperature with the primary antibodies 2H1 (1/500), anti-Flag (1/10000), anti-E-cadherin (1/250), anti-desmoplakin (1/5; Mannheim Boehringer, Germany), anti-occludin (1/250; Mannheim Boehringer, Germany), anti-ZO-1 (1/50; Zymed Laboratories Inc, CA), for 1 hour. Cells were washed 3 times in PBS and incubated for 1 hour with the appropriate secondary antibodies (1/400). Cy3- or Cy5-conjugated affinity-purified goat anti-mouse IgG or Alexa Fluor 488-conjugated goat anti-rabbit IgG were purchased from Jackson ImmunoResearch (West Grove, PA), Amersham Biosciences, and Molecular Probes (Eugene, OR), respectively. Cells were washed 3 times in PBS, and nuclei were counterstained with Hoechst 33258 dye. Slides were mounted in Vectashield (Polysciences Inc., Warrington, PA). Observation were made with a fluorescence microscope (Leica DMLB 30T, Leica Micro system, Wetzlar Germany) or confocal microscopes (Leica SP1 and Leica SP2 UV, Leica Microsystem).

### Human breast tissue analysis

Tissues were fixed in phosphate buffered formalin (4%). Immunohistochemical analysis was performed on paraffin-embedded human breast tissue sections using a peroxidase-antiperoxidase system (DAKO, Carpinteria, CA), as described previously [9]. Ten in situ carcinoma, and more than 20 samples of invasive carcinomas were studied and showed similar results.

Normal human breast epithelium studied using immunofluorescence were localized in the distal part of the paraffin-embedded biopsies of human breast carcinomas. More than 10 were tested. They were treated using the antibodies used for cell analysis (see above), as described previously [36]. Observations were made with a confocal microscopes (Leica SP1 and Leica SP2-MP, Leica Microsystem).

### Fluorescence recovery after photobleaching (FRAP)

The open reading frame of TRAF4 was cloned into the pEYFP-N1 vector (Clontech) to generate a TRAF4-EYFP fusion protein expression vector. MDCK cells were plated on glass-bottomed dishes (MatTek, Ashland, MA), transfected (JetPEI™ technic described above) with fluorescent EYFP-TRAF4. 72 hours later, the cells were incubated in a temperature controlled chamber (37°C), mounted on an inverted Leica SP2 AOBS MP microscope. Bleaching was done with an argon laser (458, 476, 488, 514 nm). Images were collected every 3.3 seconds during 150 s. Images were then averaged and the fluorescence intensity in the bleach zone was measured in the time series. The fluorescence intensity was corrected for background. The fluorescence signal was normalized to the change in total fluorescence using the following formula: Irel_t_ = (T_0_×I_t_)/(T_t_×I_0_) [37]. T_0_ total cellular fluorescence intensity during prebleach, Tt the total cellular fluorescence intensity at timepoint t, I_0_ the fluorescence intensity in the region of interest during pre-bleach and I_t_ the fluorescence intensity in the region of interest at timepoint t. Cytoplasmic fluorescence was normalized to 100 at time zero.

## Supporting Information

Movie S1 MDCK cells transfected with EYFP-TRAF4 for 72 hours were analyzed by FRAP. One representative cell expressing EYFP-TRAF4 is shown. All TJs were bleached and pictures were taken every 3.3 s. EYFP-TRAF4 rapidly recovers within the bleached area indicating that TRAF4 is a highly mobile protein.
(2.03 MB MPG)

Movie S2 MDCK cells transfected with EYFP-TRAF4 for 72 hours were analyzed by FRAP. Two representative cells expressing EYFP-TRAF4 are shown. Pictures were taken every 3.3 s. While the fluorescence intensity remains constant in the cytoplasm of the unbleached cell (upper cell) it decreases over time in the bleached cell (lower cell), showing that cytoplasmic EYFP-TRAF4 molecules are mobilized to the bleached area during recovery. Thus TRAF4 shuttles very rapidly between the cytoplasm and TJs.
(2.03 MB TIF)
