# Correction: Tumor Necrosis Factor Receptor-Associated Factor 4 Is a Dynamic Tight Junction-Related Shuttle Protein Involved in Epithelium Homeostasis

**DOI:** 10.1371/annotation/2019a487-1af6-4f4e-882a-16398799e882

**Published:** 2008-11-21

**Authors:** Valérie Kédinger, Fabien Alpy, Aurélie Baguet, Myriam Polette, Isabelle Stoll, Marie-Pierre Chenard, Catherine Tomasetto, Marie-Christine Rio

The file for Movie S2 is a duplicate of Movie S1. Please view the correct file for Movie S2 here:
